# Synthesis, anti-aging and mechanism of magnolol derivatives

**DOI:** 10.3389/fchem.2023.1180375

**Published:** 2023-05-23

**Authors:** Xinxin Pang, Li Mao, Danyang Ye, Wenqi Wang, Hongliu Yang, Xiaoxiao Fan, Yuping Yang, Zhijun Su, Tao Ma, Mingqian Sun, Yonggang Liu

**Affiliations:** ^1^ School of Chinese Materia Medica, Beijing University of Chinese Medicine, Beijing, China; ^2^ Beijing Tide Pharmaceutical Co, Ltd., Beijing Econnomi Technological Development Area (BDA), Beijing, China; ^3^ Institute of Basic Medical Sciences, Xiyuan Hospital, China Academy of Chinese Medical Sciences, Beijing, China

**Keywords:** magnolol derivatives, lifespan, stress resistant, *Caenorhabditis elegans*, magnolol

## Abstract

Magnolol (M), a hydroquinone containing an allyl side chain, is one of the major active components of *Houpoea officinalis* for antioxidation and anti-aging. To enhance the antioxidant activity of magnolol, the different sites of magnolol were structurally modified in this experiment, and a total of 12 magnolol derivatives were obtained. Based on the preliminary exploration of the anti-aging effect of magnolol derivatives in a *Caenorhabditis elegans* (*C. elegans*) model. Our results indicate that the active groups of magnolol exerting anti-aging effects were allyl groups and hydroxyl on the phenyl. Meanwhile, the anti-aging effect of the novel magnolol derivative M27 was found to be significantly superior to that of magnolol. To investigate the effect of M27 on senescence and the potential mechanism of action, we investigated the effect of M27 on senescence in *C. elegans*. In this study, we investigated the effect of M27 on *C. elegans* physiology by examining body length, body curvature and pharyngeal pumping frequency. The effect of M27 on stress resistance in *C. elegans* was explored by acute stress experiments. The mechanism of M27 anti-aging was investigated by measuring ROS content, DAF-16 nuclear translocation, sod-3 expression, and lifespan of transgenic nematodes. Our results indicate that M27 prolonged the lifespan of *C. elegans*. Meanwhile, M27 improved the healthy lifespan of *C. elegans* by improving pharyngeal pumping ability and reducing lipofuscin accumulation in *C. elegans*. M27 increased resistance to high temperature and oxidative stress in *C. elegans* by reducing ROS. M27 induced DAF-16 translocation from cytoplasm to nucleus in transgenic TJ356 nematodes and upregulated the expression of sod-3 (a gene downstream of DAF-16) in CF1553 nematodes. Furthermore, M27 did not extend the lifespan of daf-16, age-1, daf-2, and hsp-16.2 mutants. This work suggests that M27 may ameliorate aging and extend lifespan in *C. elegans* through the IIS pathway.

## Graphical Abstract

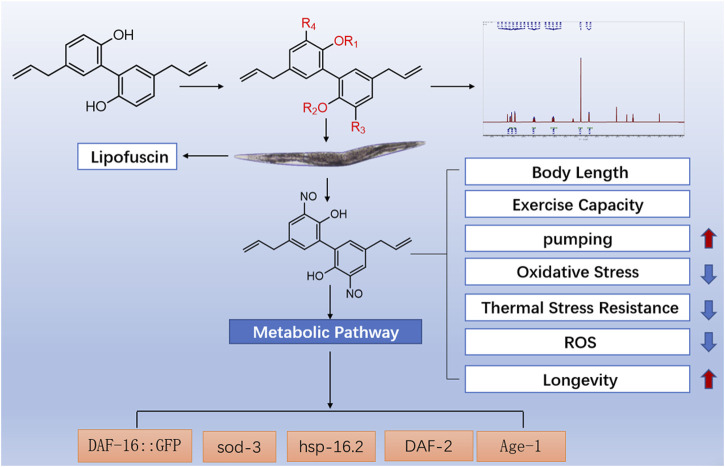

## 1 Introduction

Magnolol (M) is a natural active monomer containing allyl bisphenol isolated from *Houpoea officinalis* (Tomita et al., 1968). Its biological activities are various, including antioxidant (Subramaniyan et al., 2019), antibacterial (Liu et al., 2021a; Liu et al., 2021b), anti-inflammatory (Zhou et al., 2019), and anti-tumor (Wang et al., 2022). Magnolol is a major component of the antiaging and antioxidant properties of *Houpoea officinalis* (Zhang et al., 2019). In recent years, less attention has been paid to the structural modifications of magnolol for antioxidants and anti-aging. Studies have found that magnolol has effects on antioxidant and anti-aging because of two free phenolic hydroxyl and allyl groups. In this experiment, the addition reaction at the allyl group occurred and the hydroxyl group on the phenyl of magnolol was replaced. Additionally, it was found that the cytotoxicity to normal cells could be significantly reduced by methylation/di-methylation at hydroxyl on the phenyl of magnolol (Rempel et al., 2013; Wang et al., 2017). In our study, methyl (Zhang et al., 2022) and isopropyl (Niu et al., 2022) groups adding at the hydroxyl group on the phenyl aim to reduce toxicity and increase efficacy. In this experiment, allyl, 2-propynyl (Niu et al., 2022), and nitroso (Lin et al., 2019) with better antioxidant activity were introduced to improve the effect on anti-aging. The ester and benzyl group were also introduced to magnolol, hoping to find magnolol derivatives with better anti-aging effect. Twelve magnolol derivatives were obtained in this experiment and their anti-aging activity was explored *in vivo*. This experiment has verified the relationship between the anti-aging effect of magnolol and the free phenolic hydroxyl and allyl double bonds. The activity screening *in vivo* has revealed that the anti-aging activity of 3,3′-dinitroso magnolol (M27) is superior to that of magnolol and further explored the anti-aging mechanism of M27.

In recent years, as the aging population has gradually increased, the number of patients with diseases related to aging is on the rise (Collier et al., 2011). Physiological changes caused by aging underlie the vast majority of chronic diseases, including neurodegenerative diseases, tumors, cardiovascular diseases, and type II diabetes, among others (Movahedian et al., 2020; Sander et al., 2015; Solfrizzi et al., 2019). With the development of research on aging, there are many theories proposed for the generation of aging, including free radical aging theory, telomere theory, immunology theory, mitochondrial theory, and apoptosis(Lopez-Otin et al., 2013), and the free radical theory proposed by Denham is one of the mainstreams to explain aging in the current year (HARMAN, 1956; HARMAN, 1972). According to the free radical theory, the excess of free radicals in the body leads to mutation, deletion, or accumulation of mtDNA, which causes aging (Pomatto and Davies, 2018). Although senescence is inevitable, the process of senescence may be controlled by antioxidants. Therefore, small molecules with natural antioxidation are attracting more and more attention. More than 300 compounds with significant antioxidant activity have been reported to date, including 185 natural compounds (Ding et al., 2017; Liu et al., 2021; Zhu et al., 2020). Over the past several decades, anti-senescence research has progressed substantially and many genetic pathways related to improved aging have been identified. These pathways mainly include dietary restriction, rapamycin, and the insulin/IGF-1 signaling pathway (ⅡS), and ⅡS is the first discovered pathway who is very conserved (Zhang S et al., 2020).

*C. elegans* is a multicellular model organism, and its somatic development lineage has been studied (Zeng et al., 2021). More than 65% of the genes of *C. elegans* are homologous to humans, and their general aging characteristics in the aging process are same as that of higher organisms (He et al., 2018). Because of its short lifespan, simple physiology, and genetic traceability, *C. elegans* is an ideal model for anti-aging research and anti-aging drug screening (Li et al., 2021a). This experiment based on the *C. elegans* model aims to explore the anti-aging mechanism of M27.

## 2 Materials and methods

### 2.1 Synthesis of magnolol derivatives

**SCHEME 1:**
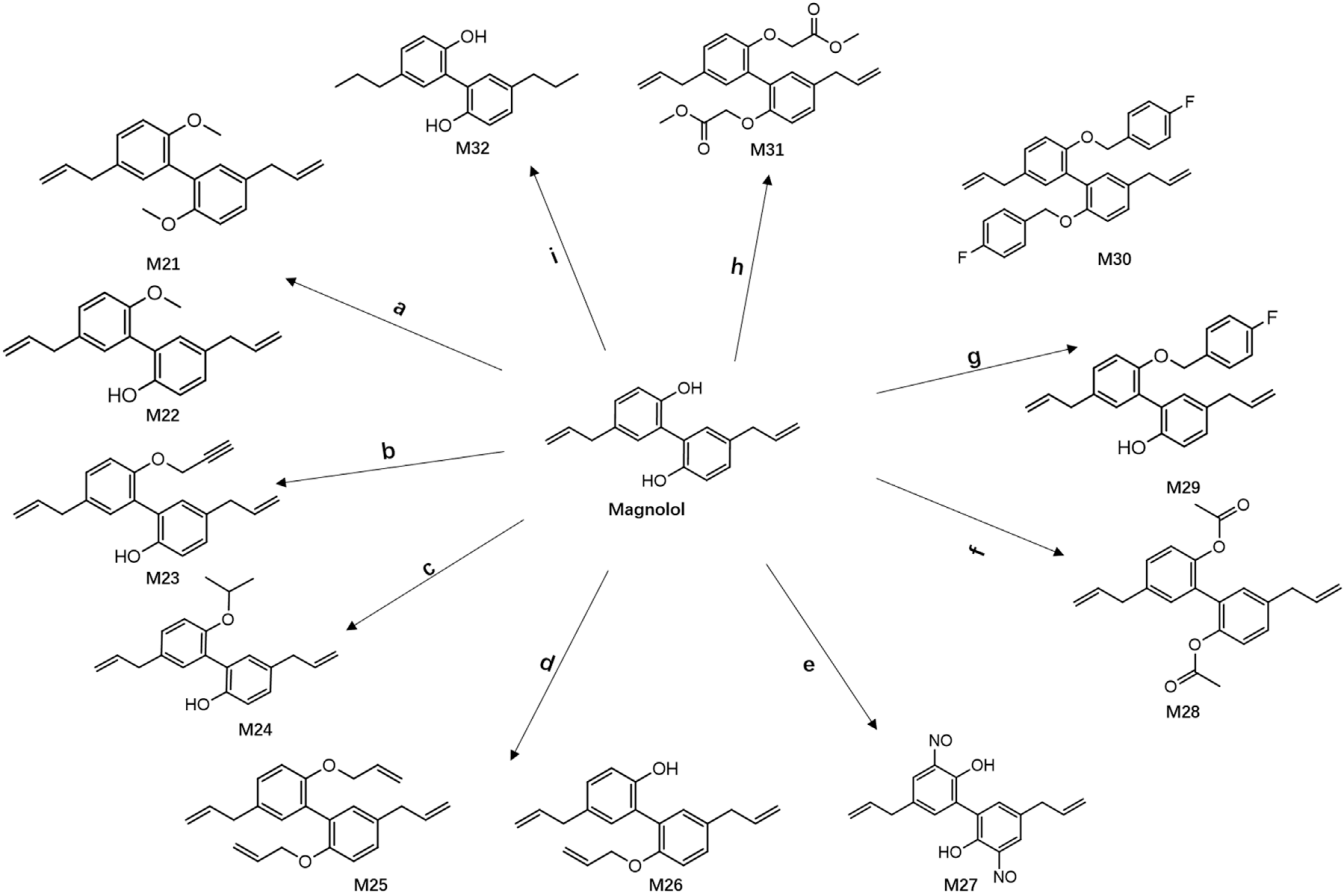
Reagents and conditions: (a) Propargyl bromide, Sodium hydride, DMF, CH_3_I, r.t; (b) Acetylene bromide,K_2_CO_3_,DMF, r.t; (c) 2-Iodopropane, K_2_CO_3_, DMF, r.t; (d) Allyl bromide, sodium hydride, DMF, CH_3_I, r.t; (e) sodium nitrite, HCl (36%), ACN/H_2_O, r.t; (f) Acetic anhydride, AC, pyridine, r.t; (g) 3-fluorobenzylchloride, K_2_CO_3_, DMF, r.t; (h) Methyl bromoacetate, K_2_CO_3_, DMF, 80°C; (i) Sodium borohydride, Nickel (ii) Chloride hexahydrate, MeOH, 0°C.

Based on the existing studies, 12 derivatives of magnolol were designed and synthesized (Scheme 1). Compounds M21-M26 and M29-M31 were synthesized by Williamson alkylation (Zhang et al., 2022). Compound M27 (Lin et al., 2019) was prepared by nitration method (Li et al., 2021b). Compound M28 was prepared by the acetylation method (Lin et al., 2016). Compound M32 was prepared by an addition reaction.

#### 2.1.1 Materials and instruments

All reagents and chemicals were purchased from China and used without further purification. ^1^H NMR spectra and ^13^C NMR were recorded on a Bruker AVANCE NEO400 MHz. The chemical shifts are given in ppm, and J-values are reported in Hz.

#### 2.1.2 2,2′-O-dimethylmagnolol M21

Compound M21 was obtained using previously reported methods (Zhang et al., 2022). Yield: 40%, colorless oil, ^1^H NMR (Chloroform-d, 400 MHz) δ 7.14 (2H, dd, J = 8.4, 2.3 Hz, H-4, H-4′), 7.06 (2H, d, J = 2.3 Hz, H-6, H-6′), 6.91 (2H, d, J = 8.4 Hz, H-3, H-3′), 5.99 (2H, ddt, J = 16.8, 10.0, 6.7 Hz, H-8, H-8′), 5.19–4.91 (4H, m, H-9, H-9′), 3.76 (6H, s, H-10, H-10′), 3.37 (4H, d, J = 6.8 Hz, H-7, H-7’) ^13^C NMR (DMSO-d6, 101 MHz) δ 155.65, 138.54, 131.60, 131.41, 128.69, 128.01, 115.94, 111.78, 55.94, and 39.09

#### 2.1.3 2-O-methyl magnolol M22

Compound M22 was obtained using previously reported methods (Zhang et al., 2022).Yield: 28%, yellow oil, ^1^H NMR (400 MHz, Chloroform-d) δ 7.20 (1H, dd, J = 8.4,2.3 Hz, H-4), 7.16 (1H, d, J = 2.3 Hz, H-6), 7.12 (1H, dd, J = 8.2, 2.3 Hz, H-4′), 7.07 (1H, d, J = 2.2 Hz, H-6′), 6.98 (1H, d, J = 5.4 Hz, H-3), 6.96 (1H,d, J = 5.2 Hz, H-3′), 6.23 (1H, s, -OH), 5.98 (2H, m, H-8, H-8′), 5.14–5.02 (4H, m, H-9, H-9′), 3.88 (3H, s, H-10), 5.98 (2 h, ddd, J = 16.9, 10.1, 6.8 Hz, H-8, H-8′), 5.14–5.08 (2H, m, H-9), 5.08–5.04 (2H, m, H-9′), 3.88 (3H, s, H-10), 3.44–3.34 (4H, m, H-7, H-7’). ^13^C NMR (DMSO-d6, 101 MHz) δ 155.60, 153.42, 138.78, 138.59, 131.69, 131.56, 131.46, 129.83, 128.49, 128.46, 128.28, 126.03, 115.92, 115.83, 115.72, 111.75, 55.89, 39.18, and 39.13

#### 2.1.4 2-O-propargyl magnolol M23

Compound M23 was obtained using previously reported methods (Niu et al., 2022). Yield: 60.5%, colorless oil, ^1^H NMR (Chloroform-d, 400 MHz) δ 7.21 (1H, dd, J = 8.4, 2.3 Hz, H-4), 7.15 (1H, d, J = 2.3 Hz, H-6), 7.12 (1H, d, J = 3.5 Hz, H-6′), 7.11–7.09 (1H, m, H-4′), 7.05 (1H, d, J = 2.2 Hz, H-3), 6.95 (1H, d, J = 8.2 Hz, H-3′), 6.04–5.91 (2H, m, H-8, H-8′), 5.21–4.98 (4H, m, H-9, H-9′), 4.69 (2H, d, J = 2.4 Hz, H-10), 3.52–3.26 (4H, m,H-7, H-7’), 2.50 (1H, s, H-12).^13^C NMR (DMSO-d6, 101 MHz) δ 153.62, 153.40, 138.71, 138.44, 132.37, 131.96, 131.66, 129.80, 128.80, 128.56, 128.29, 125.58, 115.99, 115.85, 115.72, 113.53, 80.15, 78.28, 60.22, 39.20, and 39.15.

#### 2.1.5 2-O-isopropyl magnolol M24

Compound M24 was obtained using previously reported methods (Niu et al., 2022). Yield: 75%, colorless oil, ^1^H NMR (Methanol-d4, 400 MHz) δ 7.12 (1H, dd, J = 8.2, 2.3 Hz, H-4), 7.09 (1H, d, J = 2.2 Hz, H-6), 7.02 (1H, d, J = 2.3 Hz, H-6′), 7.00 (1H, m, H-4′), 6.98 (1H, s, H-3), 6.83 (1H, d, J = 8.7 Hz, H-3′), 5.98 (2H, ddd, J = 17.0, 10.1, 2.1 Hz, H-8, H-8′), 5.13–5.05 (2H, m, H-9), 5.05–4.99 (2H, m, H-9′), 4.36 (1H, h, J = 6.1 Hz, H-10), 3.36 (2H, d, J = 6.8 Hz, H-7), 3.34–3.30 (2H, m, H-7’), 1.17 (6H, d, J = 6.1 Hz, H-11, H-12). ^13^C NMR (DMSO-d6, 101 MHz) δ 153.69, 153.25, 138.79, 138.51, 132.22, 131.98, 131.58, 129.59, 129.41, 128.40, 128.30, 125.89, 115.95, 115.88, 115.57, 115.36, 70.68, 39.37, 39.24, 22.35, and 21.18.

#### 2.1.6 2-O-Vinyl magnolol M25

Compound M25 was obtained using previously reported methods (Zhang et al., 2022).Yield: 42.5%, colorless oil.^1^H NMR (Chloroform-d, 400 MHz) δ 7.10 (2H, d, J = 2.4 Hz, H-4, H-4′), 7.07 (2H, d, J = 2.3 Hz, H-6, H-6′), 6.86 (2H, d, J = 8.1 Hz, H-3, H-3′), 6.05–5.95 (2H, m, H-11, H-11′), 5.95–5.85 (2H, m, H-8, H-8′), 5.26–5.10 (4H, m, H-12, H-12′), 5.10–5.00 (4H, m, H-9, H-9′), 4.46 (4H, dd, J = 4.7, 1.8 Hz, H-10, H-10′), 3.35 (4H, d, J = 6.7 Hz, H-7, H-7’). ^13^C NMR (DMSO-d6, 101 MHz) δ 154.45, 138.48, 134.35, 131.64, 131.62, 128.66, 128.07, 116.72, 115.96, 112.81, 68.74, and 39.13.

#### 2.1.7 2,2′-O-divinylmagnolol M26

Compound M26 was obtained using previously reported methods (Zhang et al., 2022).Yield: 25.5%, colorless oil.^1^H NMR (Chloroform-d, 400 MHz) δ 7.18 (1H, d, J = 4.4 Hz, H-4), 7.17 (1H, s, H-3), 7.12 (1H, dd, J = 8.1, 2.2 Hz, H-4′), 7.08 (1H, d, J = 2.2 Hz, H-3′), 6.98 (1H, d, J = 3.0 Hz, H-6′), 6.96 (1H, d, J = 2.2 Hz, H-6′), 6.38 (1H, s,-OH), 6.07–5.99 (1H, m, H-11), 5.99–5.91 (2H, m, H-8, H-8′), 5.48–5.19 (2H, m, H-12), 5.10 (4H, dd, J = 17.0, 1.8 Hz, H-9, H-9′), 4.58 (2H, d, J = 5.3 Hz, H-10), 3.58–3.13 (4H, m, H-7, H-7’).^13^C NMR (DMSO-d6, 101 MHz) δ 154.41, 153.40, 138.76, 138.55, 134.42, 131.83, 131.72, 131.65, 129.70, 128.50, 128.38, 125.79, 116.75, 115.96, 115.80, 115.71, 113.19, 68.78, 39.20, and 39.16.

#### 2.1.8 3,3′-dinitrosomagnolol M27

Magnolol (266 mg) and sodium nitrite (2.07 g) were dissolved in 30 mL of acetonitrile in aqueous solution (V/V = 5:1), followed by the addition of concentrated hydrochloric acid (36%, 5 mL) in an ice bath for 1 h with stirring, and the mixture was stirred for 30 min at room temperature. Yield: 89%, Yellow crystal. ^1^H NMR (Chloroform-d, 400 MHz) δ 10.86 (2H, d, J = 0.6 Hz, -OH), 8.01 (2H, d, J = 2.4 Hz, H-4, H-4′), 7.44 (2H, d, J = 2.3 Hz, H-6, H-6′), 6.01–5.89 (2H, m, H-8, H-8′), 5.18–5.11 (4H, m, H-9, H-9′), 3.42 (4H, d, J = 6.7 Hz, H-7, H-7’). ^13^C NMR (DMSO-d6, 101 MHz) δ 149.60, 138.47, 137.35, 136.34, 131.54, 128.89, 124.44, 117.18, and 38.25

#### 2.1.9 2,2′-O-diacetyl magnolol M28

Compound M28 was obtained using previously reported methods (Lin et al., 2016). Yield: 85%, White solid ^1^H NMR (DMSO-d6, 400 MHz) δ 7.24 (2H, dd, J = 8.3, 2.2 Hz, H-4, H-4′), 7.13 (2H, d, J = 8.2 Hz, H-3, H-3′), 7.06 (2H, d, J = 2.1 Hz, H-6, H-6′), 6.05–5.91 (2H, m, H-8, H-8′), 5.13–5.03 (4H, m, H-9, H-9′), 3.39 (4H, d, J = 6.8 Hz, H-7, H-7′), 1.99 (6H, s, H-11, H-11’). ^13^C NMR (DMSO-d6, 101 MHz) δ 169.39, 146.44, 137.84, 131.15, 130.21, 129.39, 123.35, 116.62, 39.17, and 20.97.

#### 2.1.10 2-O-4-fluorobenzyl magnolol M29

Compound M29 was obtained using previously reported methods (Maioli et al., 2018). Yield: 25.7%, pale yellow oils; ^1^H NMR (DMSO-d6, 400 MHz) δ 9.05 (1H, s, -OH), 7.37 (2H, dd, J = 8.5, 5.7 Hz, H-12, H-16), 7.14 (2H, d, J = 8.9 Hz, H-13, H-15), 7.08 (1H, dd, J = 8.4, 2.3 Hz, H-4), 7.02 (2H, d, J = 10.8 Hz,H-3,H-3′), 6.96–6.89 (2H, m, H-6, H-6′), 6.81 (1H, d, J = 8.0 Hz, H-4′), 6.07–5.82 (2H, m, H-8, H-8′), 5.13–5.01 (4H, m, H-9, H-9′), 4.99 (2H, s, H-10), 3.32 (2H, d, J = 6.9 Hz, H-7), 3.26 (2H, d, J = 6.8 Hz, H-7). ^13^C NMR (DMSO-d6, 101 MHz) δ 163.20, 154.40, 153.41, 138.75, 138.51, 134.21, 134.18, 131.95, 131.90, 131.72, 129.78, 129.74, 129.66, 128.70, 128.59, 128.43, 125.73, 115.99, 115.71, 115.53, 115.32, 113.46, 69.28, 39.19, and 39.17

#### 2.1.11 2,2′-O-di-4-fluorobenzyl magnolol M30

Compound M30 was obtained using previously reported methods (Maioli et al., 2018). Yield: 40.8%, White crystal; ^1^H NMR (Chloroform-d, 400 MHz) δ 7.13 (2H, s, H-12, H-12′), 7.12 (2H, d, J = 3.6 Hz, H-16, H-16′), 7.11 (2H, s, H-13, H-13′), 7.10–7.07 (2H, m, H-15, H-15′), 6.90 (2H, s, H-4, H-4′), 6.88 (2H, s, H-3, H-3′), 6.86 (1H, d, J = 2.0 Hz, H-6, H-6′), 5.96 (2H, ddt, J = 16.7, 10.0, 6.7 Hz, H-8, H-8′), 5.06 (4H, dd, J = 16.9, 1.8 Hz, H-9, H-9′), 4.90 (4H, s, H-10, H-10′), 3.35 (4H, d, J = 6.7 Hz, H-7, H-7’). ^13^C NMR (DMSO-d6, 101 MHz) δ 162.79, 154.30, 138.44, 134.03, 131.94, 131.87, 129.56, 129.47, 128.80, 128.03, 115.99, 115.54, 115.33, 113.34, 69.21, and 39.10

#### 2.1.12 2,2′-O-methyl acetate magnolol M31

Compound M31 was obtained using previously reported methods (Jia et al., 2018). Yield: 75%, White crystal 1H NMR (Chloroform-d, 400 MHz) δ 7.14 (2H, d, J = 2.3 Hz, H-6, H-6′), 7.10 (2H, dd, J = 8.3, 2.3 Hz, H-4, H-4′), 6.80 (2H, d, J = 8.3 Hz, H-3,H-3′), 5.98 (2H, ddt, J = 16.9, 10.0, 6.8 Hz, H-8, H-8′), 5.05 (4H, d, J = 9.9 Hz, H-9, H-9′), 4.56 (4H, s,H-10,H-10′), 3.73 (6H, s, H-12, H-12′), 3.37 (4H, d, J = 6.8 Hz, H-7, H-7’) .^13^C NMR (DMSO-d6, 101 MHz) δ 169.90, 154.08, 138.39, 132.60, 131.83, 128.74, 127.82, 116.08, 113.03, 65.91, 52.15, and 39.11

#### 2.1.13 5,5′- dipropyl - Resorcinol M32

Magnolol (266 mg) and nickel chloride hexahydrate (0.628 mg) were dissolved in 20 mL of anhydrous methanol, and sodium borohydride (756 mg) was added slowly with stirring in an ice salt bath. The temperature of the system do not exceed 5°C. After stirring for 0.5 h at low temperature, the mixture continued to be stirred for 1.5 h at room temperature. Yield: 34.6%, White powder 1H-NMR (Chloroform-d, 400 MHz) δ 7.12 (2H, dd, J = 8.3, 2.2 Hz, H-4, H-4′), 7.07 (2H, d, J = 2.2 Hz, H-6, H-6′), 6.95 (2H, d, J = 8.2 Hz, H-3, H-3′), 5.39 (2H, s,-OH), 2.62–2.48 (4H, m, H-7, H-7′), 1.64 (4H, dt, J = 15.0, 7.5 Hz, H-8, H-8′), 0.95 (6H, t, J = 7.3 Hz, H-9, H-9′). ^13^C NMR (DMSO-d6, 101 MHz) δ 152.67, 132.78, 131.75, 128.24, 126.24, 116.10, 36.99, 24.89, and 14.17.

### 2.2 *C. elegans* strains and culture conditions

All strains were obtained from the Genetics Center (*Caenorhabditis* Genetics Center, CGC), including N2 (wild type), CF1553 (muIs84 [(pAD76) *sod-3*::GFP)], DR26 *daf-16* (m26), TJ356 (zIs356 [daf-16p::daf-16a/b: GFP + rol-6]), VC475 *hsp-16.2* (gk249), CB1370 *daf-2* (e1370) III and TJ1052 *age-1* (hx546). In the whole experiment, all strains were maintained and grown on the nematode growth medium (NGM) plate inoculated with *E. coli* OP50.

### 2.3 Determination of body length

Nematode eggs were incubated at 20°C for 48 h to obtain synchronized wild-type L4 larvae worms. M and M27 were dissolved in *E. coli* OP50 to obtain 100 μmol·L^−1^ of M cultures and 12.5, 50, and 100 μmol·L^−1^ of M27 cultures, respectively. Synchronized L4 worms were randomly transferred to normal medium and NGM plates with M or different concentrations of M27. After 24 h incubation at 20°C, worms were picked onto slides with 1 drop of M9 solution and 1 drop of 0.5% 1-phenoxy-2-propanol solution. Images of the worms were taken with a fluorescent inverted microscope (Nikon, TS-2), and the body length of the worms was measured by NIS-Elements software. The experiment was repeated three times independently.

### 2.4 Exercise capacity measurement

Synchronized wild-type L4 worms were incubated on NGM plates same to 2.3. The body bend number of worm within 20 s was counted with microscope on the fourth and eighth day, respectively. The experiment was repeated three times independently, 20 worms per group.

### 2.5 Pharyngeal pumping assay

Synchronized L4 worms were incubated on blank and NGM plates same to 2.3. The pharyngeal pump frequency of nematode was counted under the microscope for the 20 S on days 5 and 9, respectively. The experiment was repeated three times independently, 20 nematodes per group.

### 2.6 Determination of the lipofuscin level

Synchronized wild-type L4 larvae worms were incubated on NGM plates same to 2.3 for 5 days. Worm morphology was observed in the bright and dark using a fluorescent inverted microscope (Nikon, TS -2), image of individual was taken and the fluorescence intensity was analyzed using ImageJ software. The experiment was repeated three times independently.

### 2.7 Oxidative stress assay

Synchronized wild-type L4 larvae worms were transferred to NGM plates same to 2.3 for 4 days before nematodes were transferred to new plates containing 450 μmol·L^−1^ of carob quinone. The number of dead worms was counted every 1 h until all nematodes were dead. The experiment was repeated three times independently.

### 2.8 Thermal stress resistance assay

Synchronized wild-type L4 larvae worms were transferred to blanks and NGM plates same to 2.3 and incubated for 4 days at 20°C. These nematodes were left at 35°C for 3 h, then the number of dead worms was counted for worm mortality every hour until all worms were dead. The experiment was repeated three times independently, 40 worms per group.

### 2.9 Measurement of reactive oxygen species (ROS)

Synchronized wild-type L4 larvae were transferred to an NGM medium same to 2.3. After 5 days, the worms were washed with M9 buffer and then stained with 20 μmol·L^−1^ H_2_DCFDA for 2 h at 20°C. The worms were washed with M9 to remove the dye. The worms were picked onto slides with 1 drop of M9 solution and 1 drop of 0.5% 1-phenoxy-2-propanol solution to observe their morphology in the bright and dark fields by a fluorescent inverted microscope (Nikon, TS-2). Images of individual worms were taken and the fluorescence intensity of worms were analyzed by ImageJ software.

### 2.10 Lifespan assays

At least 50 synchronized wild-type L4 larvae were transferred to NGM plates same to 2.3 for lifespan experiment. The days of transferring nematodes to the experimental plate was day 0. Death of worms was determined by mechanical stimulation. Live and dead worms were counted daily until all nematodes were dead. The nematodes were transferred to a new experimental plate each day. The experiment was repeated three times independently.

### 2.11 DAF-16 nuclear localization assays

The subcellular location of DAF-16::GFP was determined using the transgenic worms TJ356 (zls356IV). Synchronized nematode eggs were treated with M and M27. 4 days later, picture of individual was taken with a fluorescent inverted microscope (Nikon, TS-2). Worms were divided into 3 categories according to the localization of DAF-16::GFP and the number of worms in each category was counted. The experiment was repeated three times independently.

### 2.12 *Sod-3* expression in transgenic strains of *C. elegans*

Transgenic *C. elegans* strain CF1553 (muIs84) [*sod-3*::GFP] nematodes are *sod-3* and GFP fusion-expressing worms. Synchronized transgenic worm eggs were treated with M and M27 for 4 days. Images were taken by inverted fluorescence microscopy and the GFP fluorescence intensity of each group was analyzed by ImageJ software to detect the *sod-3* level in *C. elegans*. The experiment was repeated three times independently.

### 2.13 Effect of M27 on the survival of *Hsp-16.2*, *Daf-2*, *Daf-16*, and *Age-1* mutants

Synchronized transgenic L4 larvae were transferred to normal NGM plates and NGM plates with 100 μmol·L^−1^ of drug and incubated at 20°C. They were transferred to a new NGM plate each day. On day 4, they were transferred to a new plate containing 450 μmol·L^−1^ carob quinone. If the nematodes did not respond to gentle touch every 1 h, they were considered dead and the number of *C. elegans* deaths was counted. The experiment was repeated three times independently.

### 2.14 Statistical analysis

Graphs were generated using GraphPad Prism 8 (GraphPad Software Inc.), origin 2021. Lifetimes were compared using GraphPad Prism 8 (GraphPad Software Inc.). Statistical analysis was performed using SPSS 26 and results are expressed as ‾*x* ± *s*. Comparisons between multiple groups were made using one-way ANOVA if they conformed to a normal distribution with equal variance, or the Wilcoxon rank sum test if they did not conform to a normal distribution, with differences being statistically significant at *p* < 0.05.

## 3 Results and discussion

### 3.1 Anti-aging activity study of magnolol derivatives

**FIGURE 1:**
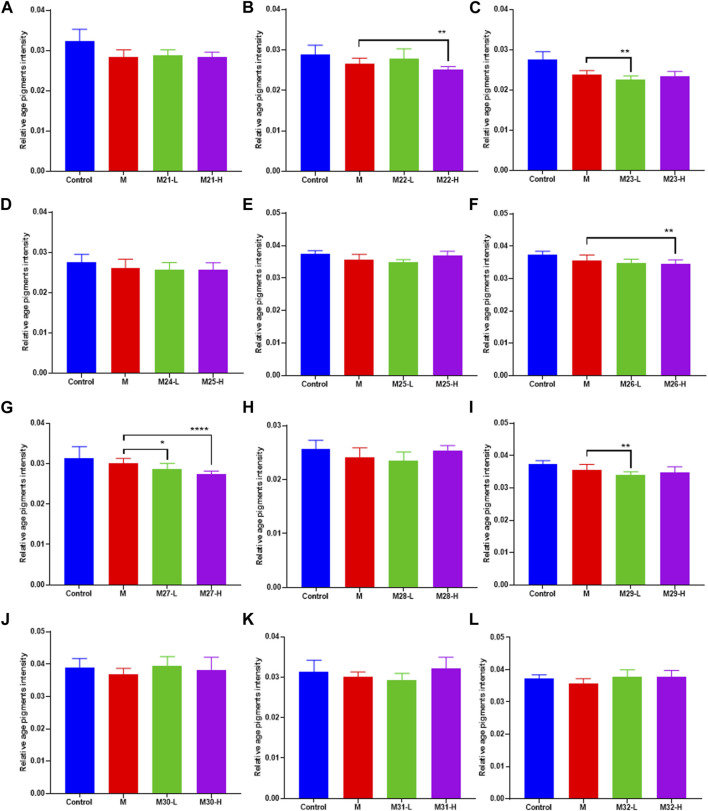
Effect of magnolol derivatives on lipofuscin levels in wild-type *C. elegans*. **(A–L)** Relative fluorescence intensity of lipofuscin accumulation in *C. elegans* after treatment with magnolol derivatives. *n* = 60, *x*‾ ± *s*. *p < 0.05, **p < 0.01, ***p < 0.001 ****p < 0.0001.

This assay evaluates the anti-aging activity of derivatives using the *C. elegans* model. Lipofuscin, an insoluble particle produced by the oxidation of unsaturated fatty acids, can reflect aging in *C. elegans* fully (Zhang X et al., 2020; Pincus et al., 2016). Lipofuscin accumulates in lysosomes and cannot be efficiently degraded. Excessive lipofuscin impairs normal protein metabolism and cellular function, which in turn accelerates nematode aging (Herndon and Driscoll, 2000). Under an inverted fluorescence microscope, blue autofluorescence of lipofuscin in *C. elegans* can be observed. In this study, the content of lipofuscin in *C. elegans* of each administration group was measured. Among these magnolol derivatives, M27 is the most effective in reducing the level of lipofuscin in *C. elegans* (Figures 1A-L) (*p* < 0.0001). According to the experimental results, we found that most of the dominant compounds were mono-substituents. The possible reason is that the formation of intramolecular hydrogen bond of magnolol is effectively reduced by mono-substitution on the phenolic hydroxyl group. In addition, mono methyl, mono allyl, and mono propynyl groups could all significantly increase the anti-aging effect of magnolol, the probably reason is that chain hydrocarbyl groups reduced the toxicity of magnolol. The 3,3′-dinitrosomagnolol was the most effective derivative, probably because of the excellent antioxidant activity of the nitroso group. However, when the two phenolic hydroxyl groups were substituted or the allyl group was added, the anti-aging activity of magnolol was significantly reduced, indicating that phenolic hydroxyl group and the allyl group play an irreplaceable role in the anti-aging activity of magnolol.

### 3.2 Effects of M27 on the healthy lifespan of *C. elegans*

**FIGURE 2:**
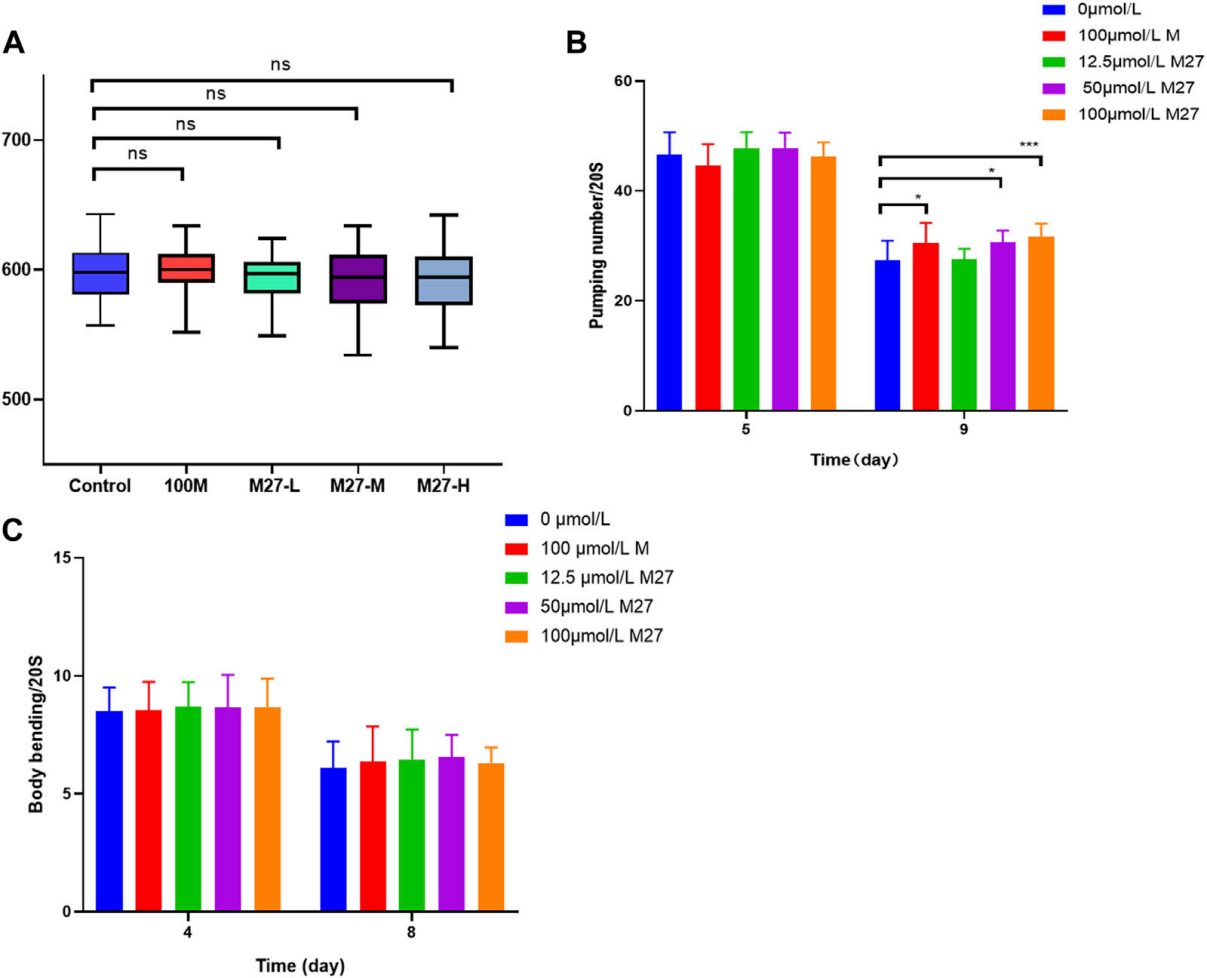
Effect of M27 on nematode healthy lifespan **(A)** Effect of M27 on body length of N2 nematodes; **(B)** Effect of M27 on locomotor activity of N2 nematodes; **(C)** Effect of M27 on pharyngeal pumping rate of N2 nematodes. *n* = 20, *x*‾ ± *s*.

The change in body length reflects the growth and development status of nematodes and the toxicity of drugs. Toxic substance will shorten the body length of nematodes (Yin, 1996). During normal aging, muscle strength and coordination decline in most animals. As they age, the number of body bends and swallows per unit of time decreases (Shi et al., 2021). We examined the effect of various concentrations of M27 on *C. elegans* body length. The nematode body length of treated groups was not significantly different from that of the blank control (Figure 2A). The motility and pharyngeal pumping rate of nematodes were measured separately. M27 had no effect on nematode motility, compared with the control group (Figure 2B). M27-M and M27-H significantly improved the pharyngeal pumping rate of *C. elegans* which declined with age (Figure 2B). The decline in muscle capacity and coordination could be partially improved by M27 treatment. The above experimental results show that M27 has no effect on the growth and development of *C. elegans* and no toxic to *C. elegans*, and can partially improve its healthy lifespan.

### 3.3 M27 improves survival of *C. elegans* under acute stress

**FIGURE 3:**
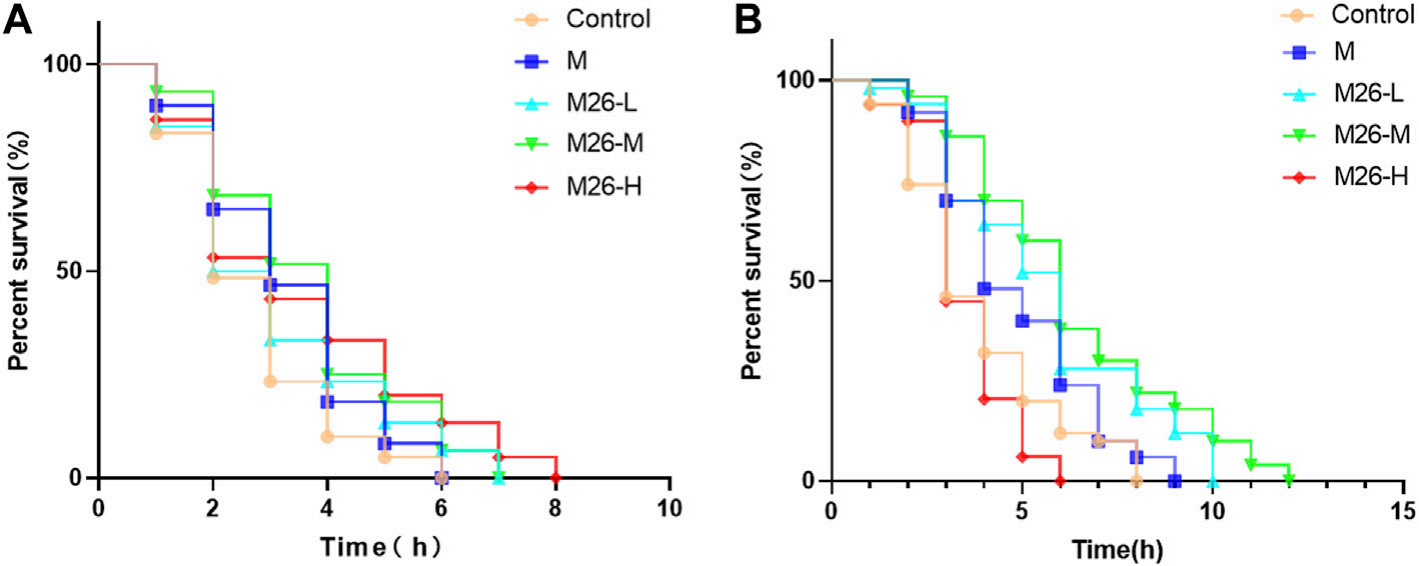
Survival curves of *C. elegans* under acute stress after M27 treatment. **(A)** Survival curves of *C. elegans* under high-temperature stress (35°C) stimulation after treatment with M (100 μmol·L^−1^) and M27 (12.5 μmol·L^−1^, 50 μmol·L^−1^, 100 μmol·L^−1^); **(B)** Survival curves of *C. elegans* after treatment with M (100 μmol·L^−1^), M27 (12.5 μmol·L^−1^, 50 μmol·L^−1^, 100 μmol·L^−1^) stimulated with 430 μmol·L^−1^ carob quinone. *n* = 40, *x*‾ ± *s*.

As ages, the nematode’s resistance to external stressful environments diminishes. Heat stress and oxidative stress have been shown to accelerate cellular oxidation in organisms (Jeon and Cha, 2016), causing high-intensity oxidative damage to *C. elegans* in a short period, expediting worm senescence. In the heat stress experiment (Figure 3A), the median lifespan of *C. elegans* in different groups was 2 h, 3 h, 2.5 h, 3 h, and 3 h. The survival curve shifted significantly to the right in the M27-H group compared with other groups, meaning that M27-H significantly extending the lifespan of *C. elegans* under heat emergency. The median lifespan of *C. elegans* in each group was 3 h, 4 h, 4.5 h, 6 h, and 6 h in the carob quinone-induced oxidative stress assay (Figure 3B). All the above results indicated that M27 and M could significantly improve the ability of *C. elegans* to resist stress under acute stress, and the effect of M27 has a significantly better than that of M, further indicating that M27 has a superior antioxidant.

### 3.4 M27 reduces intracellular ROS accumulation in *C. elegans*

**FIGURE 4:**
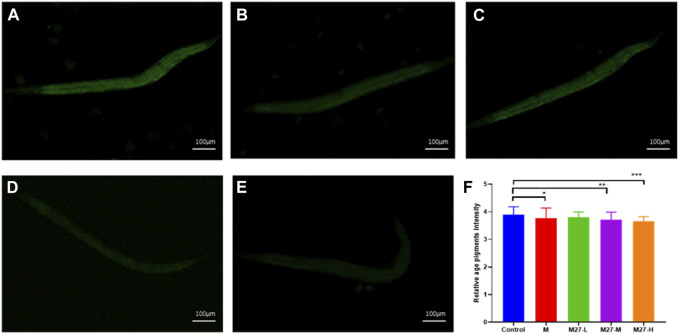
ROS levels in *C. elegans* after M27 treatment. **(A–E)** ROS levels in control, M (100 μmol·L^−1^), M27-L (12.5 μmol·L^−1^), M27-M (50 μmol·L^−1^), and M27-H (100 μmol·L^−1^) groups; **(F)** Relative fluorescence intensity of intracellular ROS in *C. elegans* from control, M, M27-L, M27-M, and M27-H groups. *n* = 30, *x*‾ ± *s*. *p < 0.05, **p < 0.01, ****p < 0.0001.

The above results suggest that M27 significantly improves the resistance of *C. elegans*, but the mechanism is not clear. Natural antioxidants cooperate with endogenous antioxidants to increase the defense against ROS and restore the proper balance by neutralizing ROS. The fluorescent probe H_2_DCFDA can be used to determine ROS levels in *C. elegans*. In this experiment, the levels of ROS in *C. elegans* were significantly reduced in M, M27-M, and M27-H groups compared to the blank group (Figure 4F), and fluorescence intensity of M27-H group is the lowest (*p* < 0.0001) (Figures 4A–F). It indicated that M27 improved stress resistance of *C. elegans* may be associated with reducing ROS level in *C. elegans*.

### 3.5 M27 extends the lifespan of *C. elegans*

**FIGURE 5:**
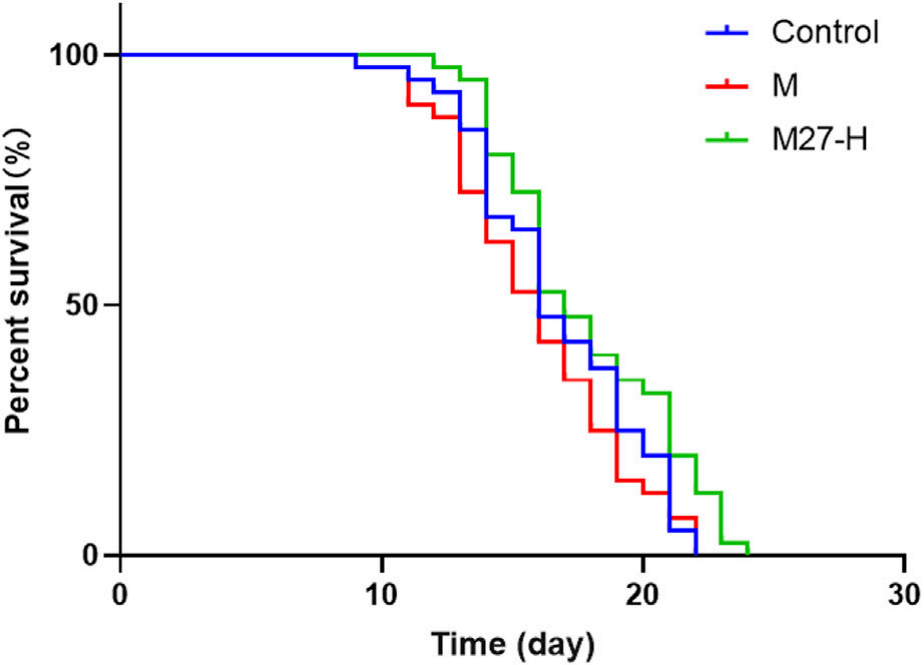
Effect of M27 on the longevity of *C. elegans*. *n* = 40, *x*‾ ± *s*. *p < 0.05.

The above results showed that M27-H was the most effective, therefore this concentration was chosen for subsequent experiments. To investigate whether M27 could prolong the lifespan of *C. elegans*, in this study, wild-type *C. elegans* was used as a model. As shown in Figure 5, the survival curve of M27-H shifted significantly to the right compared to the blank group (*p* < 0.05), which showed that M27 could extend the lifespan of *C. elegans.*

### 3.6 M27 induces nuclear translocation of DAF-16::GFP and expression of *sod-3*

**FIGURE 6:**
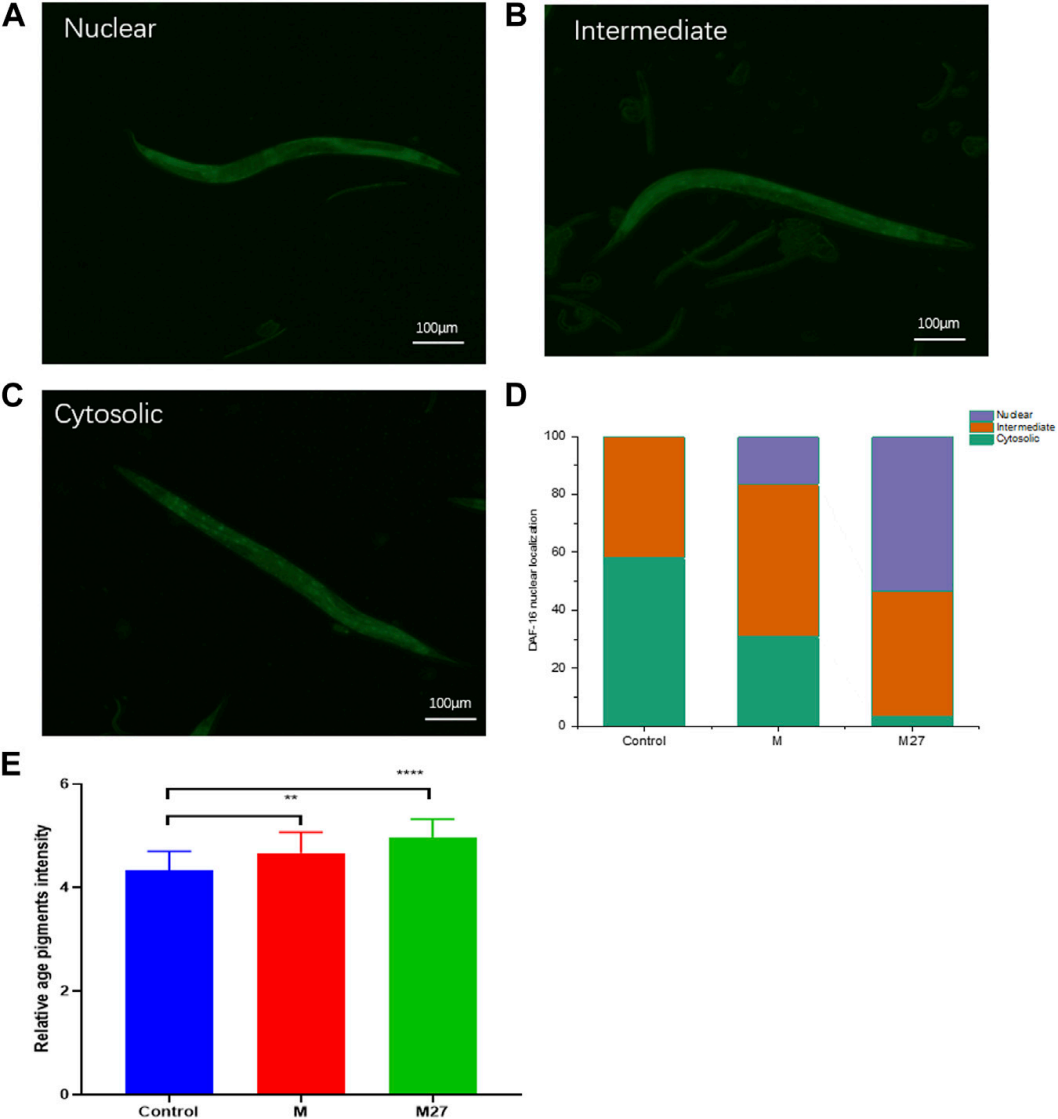
M27 induces nuclear localization of DAF-16::GFP. **(A–C)** Three expression patterns of DAF-16 in *C. elegans*; **(D)** Proportion of nuclear translocation of DAF-16 induced by M27 in TJ356 worms; **(E)** Effect of M27 on the fluorescent expression of CF1553 *worms*. *n* = 40, *x*‾ ± *s*.**p < 0.01, ***p < 0.001.

TJ356 nematodes are DAF-16 and GFP fusion-expressing nematodes. DAF-16 is essential for dauer phase formation, lifespan extension, and stress resistance in nematodes. DAF-16 is repressed by the insulin signaling pathway (Yin, 1996b). Under normal growth conditions, DAF-16 of TJ356 worms is mainly in the cytoplasm (Figure 6A). M27 was able to promote the transfer of DAF-16 from the cytoplasm to the nucleus (Figures 6B,C). M27 could significantly activate DAF-16 translocation to the nucleus (Figure 6D). Entry of DAF-16 into the nucleus activates the expression of downstream gene, *sod-3*. *sod-3* gene encodes nematode superoxide dismutase (SOD), which is involved in oxidative and antioxidant homeostasis *in vivo*. CF1553 worms are *sod-3* and GFP fusion-expressing nematodes. In this experiment, the expression of *sod-3* in nematodes was detected by observing the GFP fluorescence intensity using inverted fluorescence microscopy. The expression of *sod-3* was significantly upregulated after M27-H treatment compared to the control (Figure 6E). M27 significantly enhanced the expression of anti-oxidative stress genes. The above experimental results indicate that the anti-aging mechanism of M27 is closely related to DAF-16 and *sod-3*.

### 3.7 M27 ameliorates senescence in *C. elegans* through activation of stress response signaling pathways

**FIGURE 7:**
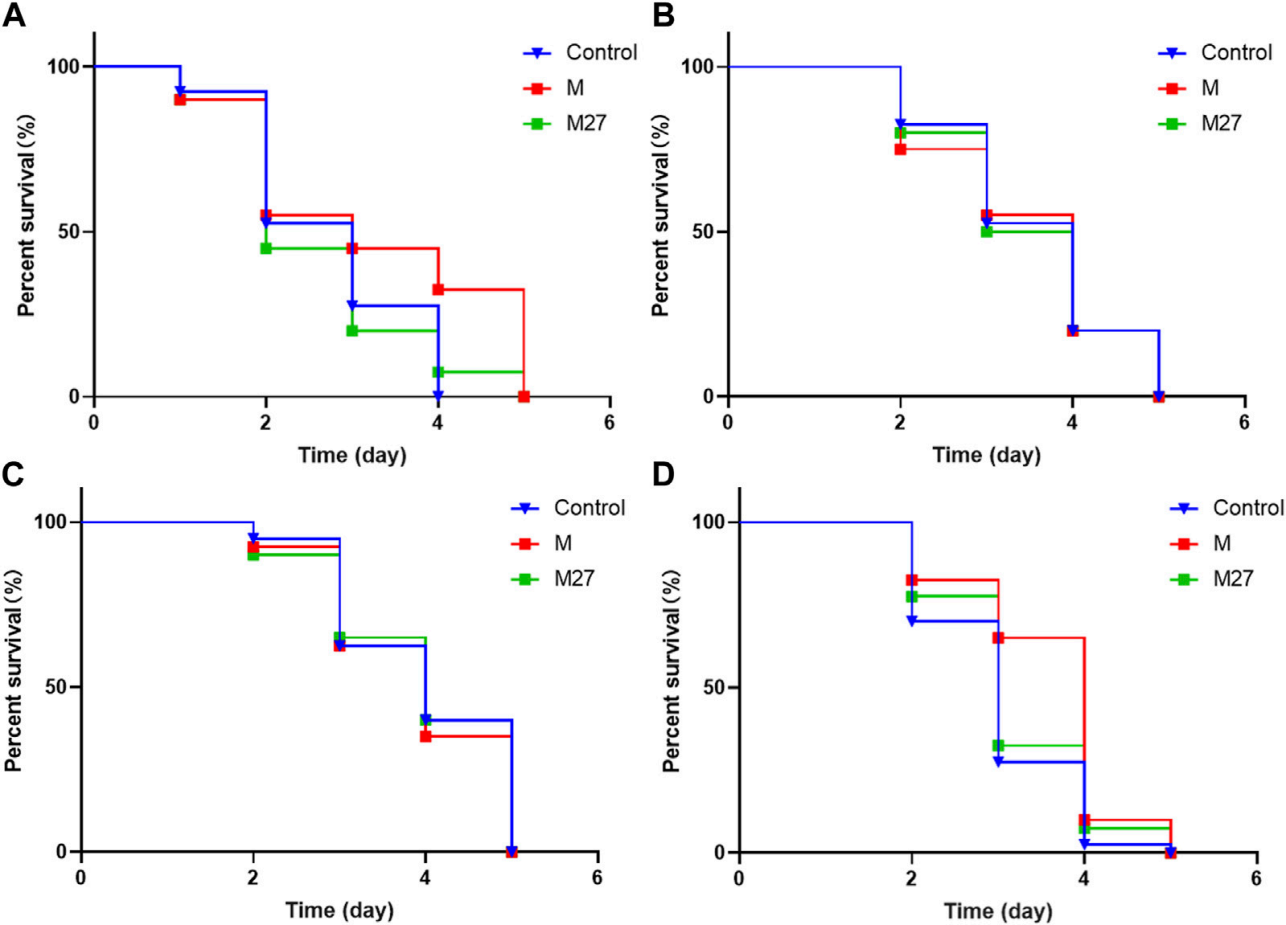
Effect of M27 on survival of loss-of-function mutant strains. **(A)** *Hsp-16.2*, **(B)** *Age-1*, **(C)** *Daf-2*, **(D)** *Daf-16* mutant worms. *n* = 40, *x*‾ ± *s*.

Downstream targets of DAF-16 include antioxidant genes such as *sod-3* and small molecule heat shock protein gene (*hsp-16.2*). Overexpression of these gene can effectively prolong the lifespan of worms and improve their ability to resist external stress. M27 could significantly increase the survival rate of N2 worms suffering from heat stress. This may be due to the upregulation of *hsp-16.2* expression by M27 increases stress resistance of worms. As shown in the figure, M27 did not improve survival after oxidative stress in *hsp-16.2* (gk249) worms (Figure 7A). The results suggest that M27 prolongs the lifespan of *C. elegans* through the activation of stress response signaling pathways.

The insulin/insulin-like pathway (IIS) is associated with lifespan, stress tolerance, metabolic regulation, protein homeostasis, and so on (Cohen and Dillin, 2008). DAF-2/IGF-1R, AGE-1/PI3K, and DAF-16/FOXO are the three main upstream, midstream, and downstream nodes of this pathway, respectively. They are commonly used as gene-related indicators to determine the antioxidant and anti-aging activity of *C. elegans* (Fei et al., 2017). In order to understand whether M27 improves the aging of *C. elegans* through the IIS pathway, oxidative stress assays were performed using *age-1* (hx546), *daf-2* (e1370), and *daf-16* (m26) worms, respectively. It was found that M27 did not improve the survival of *age-1* (hx546), *daf-2* (e1370), and *daf-16* (m26) worms under Juglone-induced oxidative stress (Figures 7B–D). This suggests that *age-1*, *daf-2*, and *daf-16* are required for M27 mediated delayed senescence in *C. elegans*. The above experimental results illustrate that M27 may exert anti-aging effects through the insulin signaling pathway.

## 4 Conclusion

According to reports, the natural product magnolol has various biological activities such as antioxidant, anti-inflammatory and anti-tumor. To improve the anti-aging activity of magnolol, a series of derivatives were designed and synthesized in this study. Through the evaluation of the activity of the derivative, it was found that M27 has the best anti-aging effect, and its effect was better than that of magnolol. It is the first time to verify the antioxidant and anti-aging activities of M27 by the *C. elegans* model system.

This study showed that M27 can extend the lifespan and reduce the accumulation of lipofuscin in *C. elegans*. M27 can significantly increase stress resistance and SOD activity and reduce ROS levels *in vivo* in *C. elegan*s. M27 significantly activates the translocation of DAF-16 to the nucleus, which activates the expression of *sod-3* and *hsp-16.2*. In addition, it was found that the anti-aging mechanism of M27 could be associated with *age-1*, *daf-2*, and *daf-16*, suggesting that M27 may improve aging and prolong the lifespan of *C. elegans* through the IIS pathway. But the effect of compound M27 on the expression of genes (*daf-2*, *age-1*, *hsp-16*) on this pathway, needs to be further examined by RT-PCR. In addition, based on the experimental results, it can be found that the lifespan of nematodes in the magnolol group was shorter than that in the blank group, and it is speculated that magnolol may have some chronic toxicity. However, when magnolol was derivatized, the lifespan of the nematode was extended, indicating that the toxicity of magnolol derivatives is significantly reduced compared with that of magnolol, which can be further examined by a later toxicity experiment.

## Data Availability

The original contributions presented in the study are included in the article, further inquiries can be directed to the corresponding authors.
